# Bacterial and fungal core microbiomes associated with small grain silages during ensiling and aerobic spoilage

**DOI:** 10.1186/s12866-017-0947-0

**Published:** 2017-03-03

**Authors:** Lysiane Duniere, Shanwei Xu, Jin Long, Chijioke Elekwachi, Yuxi Wang, Kelly Turkington, Robert Forster, Tim A. McAllister

**Affiliations:** 1 Agriculture and Agri-Food Canada Research Centre, Lethbridge, T1J 4B1 AB Canada; 2 Agriculture and Agri-Food Canada (AAFC), Lacombe, T4L 1 W1 AB Canada

**Keywords:** Barley, Oats, Triticale, Silage, Microbiome, Bacteria, Fungi, Aerobic exposure

## Abstract

**Background:**

Describing the microbial populations present in small grain silage and understanding their changes during ensiling is of interest for improving the nutrient value of these important forage crops. Barley, oat and triticale forages as well as an intercropped mixture of the 3 crops were harvested and ensiled in mini silos for a period of 90 days, followed by 14 days of aerobic exposure. Changes in fermentation characteristics and nutritive value were assessed in terminal silages and bacterial and fungal communities during ensiling and aerobic exposure were described using 16S and 18S rDNA sequencing, respectively.

**Results:**

All small grain silages exhibited chemical traits that were associated with well ensiled forages, such as low pH value (4.09 ± 0.28) and high levels of lactic acid (59.8 ± 14.59 mg/g DM). The number of microbial core genome operational taxonomic units (OTUs) decreased with time of ensiling. Taxonomic bacterial community profiles were dominated by the Lactobacillales after fermentation, with a notable increase in Bacillales as a result of aerobic exposure. Diversity of the fungal core microbiome was shown to also be reduced during ensiling. Operational taxonomic units assigned to filamentous fungi were found in the core microbiome at ensiling and after aerobic exposure, whereas the Saccharomycetales were the dominate yeast population after 90 days of ensiling and aerobic exposure. Bacterial and fungal orders typically associated with silage spoilage were identified in the core microbiome after aerobic exposure.

**Conclusion:**

Next Generation Sequencing was successfully used to describe bacterial communities and the first record of fungal communities throughout the process of ensiling and utilization. Adequately describing the microbial ecology of silages could lead to improved ensiling practices and the selection of silage inoculants that act synergistically with the natural forage microbiome.

**Electronic supplementary material:**

The online version of this article (doi:10.1186/s12866-017-0947-0) contains supplementary material, which is available to authorized users.

## Background

Silage production is of great economic importance in the world. The land area devoted to worldwide forage production was estimated at 135.7 million ha in 2000 [1]. Corn and alfalfa are the most important crops used for ensiling worldwide [2], but small grain cereals (e.g., barley, triticale, oats) and various legumes are often ensiled in Northern climates [3–5]. The type of crop selected for forage and silage production depends on their growth requirements, yield and their nutritive value [6].

Silage conservation of moist crops depends on microbial fermentation [7] with lactic acid bacteria (LAB) being the main producers of the organic acids essential for silage conservation [8, 9]. Inoculants have been selected for their ability to improve fermentation during ensiling of the forage, increase aerobic stability and enhance silage digestibility [10]. Among the many microbial species found in silage, some are considered undesirable as their presence can negatively impact silage quality. Undesirable microorganisms can lower the dry matter (DM) and water-soluble carbohydrates (WSC) content of silage [11] and cause undesirable fermentation profiles such as the production of butyric acid [12]. Among undesirable microorganisms, some such as *Listeria* sp. or mycotoxigenic fungi can be pathogenic or produce toxins that have adverse effects on the health of both livestock and humans [13]. Yeasts are the main microorganisms involved in the spoilage of silage as they metabolize lactic acid, increasing silage pH and creating conditions that are conducive for the growth of other spoilage or pathogenic microorganisms [14]. A better understanding of the microbial communities actively involved in the ensiling process could provide additional insight into approaches to improve the conservation of silages.

Many of the microorganisms found in silage such as *Lactobacillus* sp. or *Saccharomyces* sp. have been proven to enter a viable, but nonculturable state in the face of environmental stress [15, 16]. Microbial communities described solely on the basis of culturing are often incomplete as many species are unculturable or poorly represented by the culturing process. Metagenomic deep sequencing of microbial DNA is a culture-independent technique that allows microbial diversity to be described without the need to culture isolates. Ribosomal DNA (rDNA) gene sequencing has been successfully used in many studies to describe microbiomes in complex environments including the rhizosphere [17, 18], soil [19, 20], compost [21] and rumen [22]. Eikmeyer et al. [23] were the first to study the bacterial microbiome of grass silage during ensiling with and without inoculants. However, these authors focused only on bacterial communities and did not describe the nature of the fungal microbiome.

This study aimed to characterize the bacterial and fungal core microbiomes associated with small grain cereals (i.e., barley, oats, triticale) during ensiling and upon aerobic exposure.

## Methods

### Forage

Whole-crop barley (*Hordeum vulgare*, L. Variety “Sundre” [24]), oats (*Avena sativa* L. Variety “AC Morgan”, [25]) and triticale (*Triticosecale Wittm* Variety “Bunker”, [26]) or an intercropped mixture of all 3 crops were planted on 12 June 2013 at the Lacombe Research Centre, Agriculture and Agri-Food Canada (113.7° W, 52.5°N) and harvested on 4 September 2013. Individual crop species were seeded at 300 seeds m^−2^ and an intercropped mixture of the three cereals was seeded at 100 seeds m^−2^. Seeding was conducted with a 3.7 m seed drill (ConservaPak™, John Deere, Moline, IL, USA) configured with knife openers spaced 23 cm apart and a plot size of 3.7 × 7.6 m. All plots received N, P and K fertilizers applied at the time of seeding, alone or as a blend, typically in the form of urea (46-0-0), monoammonium phosphate (11-51-0), and potassium chloride (0-0-60) at rates according to soil test recommendations. Prior to seeding, the plot areas were sprayed with glyphosate [N-(phosphonomethyl)glycine] across the entirety of each site 24 to 48 h prior to seeding using label recommended rates and application parameters for the Canadian Prairies [27]. In-crop herbicides were used for weed control depending on the weed spectrum present. At harvest, crops were ensiled at DM levels of 48.1% (firm dough), 30.2% (early dough), 41.7 and 38.5% (medium dough) in barley, oats, triticale and the intercropped mixture, respectively. Forage was chopped to a theoretical chop length of 9.5 mm using a self-propelled forage harvester (Harvester 6610, John Deere, Moline, IL, USA).

### Mini silo experiment

Forages were packed into mini PVC silos (2.5 to 3 kg of fresh forage) with a hydraulic press to a density of approximately 240 kg/m^3^ as previously described [10]. The silos were weighed prior to filling and immediately after sealing, and stored at ambient temperature (22 °C). Each crop was harvested without wilting from 3 replicate field plots. Triplicate silos for each crop (one from each plot) were prepared and opened after 90 day of ensiling. Prior to ensiling (day 0), samples of each forage type from each plot were collected for chemical and microbial analyses. Silos were weighed prior to opening to calculate DM loss. At sampling, triplicate mini silos were opened and the contents were thoroughly mixed by hand. Subsamples were then collected for chemical, microbial and molecular analyses.

### Aerobic stability

After 90 days of ensiling, silos were opened and approximately 7.5% of the wet weight in each silo was subsampled. These samples were placed into separate 4-L insulated containers (3 replicates per treatment), covered with two layers of cheesecloth and stored at 20 °C for 14 days. Two Dallas Thermochron iButtons (Embedded Data Systems, Lawrenceburg, KY, USA) were embedded in the silage near the bottom and centre of each container with the temperature in the storage room recorded every 15 min. Ambient temperature and the temperature in each container were simultaneously monitored for 14 days. The contents of each container were thoroughly mixed and sampled after 14 days of aerobic exposure for chemical (15 g) and microbial analyses (10 g) and for DNA extraction (30 g). Aerobic stability was calculated according to Teller et al. [28] as the number of hours before the temperature of aerobically exposed silage exceeded the baseline ambient temperature by 2 °C.

### Chemical analysis

Forages collected at harvest and silage samples collected on d 90 from each silo were analyzed for water-soluble carbohydrates (WSC), ammonia nitrogen (NH_3_-N) and starch, as described by Zahiroddini et al. [29]. Volatile fatty acids and lactate were determined by the methods of Kudo et al. [30] on a Hewlett Packard model 5890A Series Plus II gas liquid chromatograph (column: 30 m FFAP fused silica capillary, 0.32 mm i.d., 1.0 m film thickness, Phenomenex, Torrance, CA, USA). Total nitrogen (N) was determined by elemental analysis (Dumas Nitrogen) using a NA1500 Nitrogen/Carbon analyzer (Carlo Erba Instruments, Milan, Italy). Crude protein was calculated as N × 6.25. The DM of forages and silage samples was determined by drying at 105 °C in forced-draft oven for 24 h. Organic matter (OM) was estimated by ashing 1 g of dried sample in a muffle furnace at 550 °C for 5 h. Neutral detergent fibre (NDF) and acid detergent fibre (ADF) were analyzed using an Ankom 200 system following the manufacturer’s instructions (Ankom Technology Corporation, Fairport, NY, USA) with sodium sulfite and α-amylase used for NDF analysis. Nitrogen in ADF residues (ADF insoluble nitrogen, ADIN) was measured by combustion as described above. Samples (15 g) from each mini silo at each sampling time were mixed with 135 mL of deionized water, blended for 30 s and filtered through two layers of cheesecloth and pH of the filtrate was measured with a Symphony pH meter (VWR, Mississauga, ON, Canada).

### Microbial analysis

For microbial analyses, forage or silage samples (10 g) from each mini silo were treated as described previously by Addah et al. [31] with lactobacilli counts estimated using Man Rogosa Sharpe (MRS, [32]) plates amended with 200 μg/mL of cycloheximide (MRS; Dalynn Biologicals, Calgary, AB, Canada), total bacteria using nutrient agar (NA) amended with 200 μg/mL of cycloheximide (Dalynn Biologicals, Calgary, AB, Canada) and yeast and molds using Sabouraud’s dextrose agar (SDA; Dalynn Biologicals, Calgary, AB, Canada) containing 100 μg/mL of tetracycline and 100 μg/mL of chloramphenicol. Lactobacilli MRS agar and NA plates were incubated at 37 °C for 24–48 h whereas SDA plates were incubated at ambient temperature for 48–72 h. Numbers of yeasts and filamentous fungi (i.e., molds) were differentiated based on colony appearance and morphology on SDA plates.

### Molecular analysis

#### DNA extraction

For each sampling, forage and silage samples (30 g) from each mini silo were frozen (−80 °C), lyophilized and ground through a 4 mm screen. Subsamples (5 g) were then ball milled for 1 min at ambient temperature and DNA extraction was performed according to the procedure of Yu and Morrison (2004) [33]. Briefly, DNA was extracted from 0.3 g of ground silage using a bead beating step (FastPrep-24, MP Biomedicals, Santa Ana, CA, USA; 3 min, maximum speed) with zirconia silica beads (0.3 g of 0.1 mm and 0.1 g of 0.5 mm). Nucleic acids were precipitated with ammonium acetate and isopropanol, washed in ethanol and re-suspended in Tris-EDTA buffer. All samples were purified through a QiaAmp DNA Stool kit column (Qiagen Sciences, Germantown, MD, USA) and eluted in nuclease free water. Yield and purity of extracted DNA was measured using a PicoGreen^®^ dsDNA quantitation assay (Invitrogen Canada Inc., Burlington, ON, Canada). High molecular weight DNA samples at a minimal concentration of 20 ng/μl were used for sequencing.

#### Sequencing

Extracted DNA samples were pair-end sequenced using Illumina MiSeq (San Fransico, CA, USA) at Genome Quebec (McGill University, Génome Québec Innovation Centre, Montreal, QC, Canada). For bacteria, the V3-V4 region of 16S was targeted using universal primers 347 F-CS1 (ACACTGACGACATGGTTCTACAGGAGGCAGCAGTRRGGAAT) and 803R-CS2 (TACGGTAGCAGAGACTTGGTCTCTACCRGGGTATCTAATCC). Primers nu-SSU-0817 (TTAGCATGGAATAATRRAATAGGA) and nu-SS-1196 (TCTGGACCTGGTGAGTTTCC) originally designed by Borneman and Hartin (2000) [34] were used to target around 400 bp of the genomic region containing part of the V4 and total V5 variable domains of the SSU rDNA gene from all four major phyla of fungi. Library construction and the Illumina MiSeq paired-end sequencing was performed by Genome Quebec according to manufacturer’s recommendations.

### Bioinformatic analysis

Bacterial sequences were scanned for contaminants and unpaired reads, trimmed to a fixed length of 250 bp and paired-end assembled using FLASH software [35]. Read counts per sample were checked to confirm no sample failed to be amplified or sequenced. Reads were clustered at 97% identity using DNACLUST [36] and clusters showing abundances higher than 3 were then scanned for chimeras with UCHIME denovo and UCHIME reference in order to estimate final operational taxonomic units (OTUs) [37]. OTUs were analyzed for taxonomic distribution with the RDP classifier software using the complete Greengenes database (http://greengenes.secondgenome.com/downloads) supplemented with eukaryotic sequences from the Silva databases and a customized set of mitochondria and chloroplasts 16S sequences. Each OTUs taxonomic depth with a RDP classifier score below 0.5 was kept to reconstruct the final lineage. Diversity metrics were obtained by aligning OTU sequences to a Greengenes core reference alignment [38] using the PyNAST aligner [39]. Alpha diversity index (Chao1, Shannon-Weiner indexes, and rarefaction curves), and taxonomic classifications were then computed using QIIME software [40, 41].

Fungal sequences were analyzed following the MiSeq standard operating procedure for MOTHUR analysis [42] using a process adapted for 18S rDNA gene sequences. Reference and taxonomy alignments were performed using the updated SILVA reference file v119 [43], containing the full length sequences and taxonomic references for 51,533 SSU sequences from Eukaryota, 464,618 sequences from Bacteria and 18,797 sequences from Archaea. Briefly, the reads from each sample were randomly subsampled down to 15,000 using HTSeq and Python [44]. The sequences were then assembled, trimmed and singletons were removed. Reads with homopolymers greater than eight bases and sequences with one or more ambiguous bases were removed from the data set. Remaining sequences were aligned and run against the SILVA reference database. Before being classified into OTUs, sequences were pre-clustered at 97% identity and chimeras were scanned and removed using UCHIME [37]. Final OTUs were obtained and analyzed for taxonomic distribution with the SILVA database. Alpha diversity indexes and taxonomic classifications were then computed in a manner similar to that of bacterial sequences.

Determination of the core microbiome was accomplished by comparing samples from all crops across time. For each time, any taxa found to be ubiquitous across all samples were then defined to be part of the core microbiome of small grain cereal silages.

### Calculations and statistical analysis

Microbial populations were estimated as colony forming unit (CFU)/g of forage or silage DM and were log transformed prior to statistical analysis. Ensiling parameters, aerobic stability and microbial data were assessed using a randomized complete block design considering 4 crops (barley, oats, triticale and intercrop) at 3 times of sampling (0, 90 and 104 days). An analysis of variance using the crops and time was performed to determine significant differences in diversity indexes. Significance was defined as a *P* value of less than 0.05 using SAS [45].

## Results

### Silages characteristics

At ensiling, barley forage exhibited the highest (*P* < 0.001) initial pH of all forages (Table 1). A higher level of DM was observed in barley silage compared to oat and triticale silages with the DM of intercropped silage being close to the average DM of the 3 crops. With the exception of barley, the pH of all silages rapidly declined to below 4.0 within 7 days (data not shown) of ensiling and remained stable until sampling of terminal silage (90 days). In this study, barley silage was the exception to this pattern as it gradually declined to a pH of 4.5 over 90 days of ensiling. After ensiling, DM was higher for barley silage and lower for oat silage as compared to triticale or intercropped silage (*P* < 0.001, Table 1). DM loss was significantly higher for triticale than other silages (*P* < 0.05)

**Table 1:** Physico-chemical characteristics and microbial populations of barley, oat, triticale and intercropped forages, 90day silages and aerobically exposed silages (*n* = 3)

Item	Forage				SEM	*P*-value^*^	Silage				SEM	*P*-value ^*^	Aerobically exposed Silage				SEM	*P*-value^*^
	Barley	Oat	Triticale	Intercropped			Barley	Oat	Triticale	Intercropped			Barley	Oat	Triticale	Intercropped		
pH	7.57^b^	6.28^d^	6.52^c^	6.57^c^	0.143	<0.001	4.50^b^	3.98^c^	3.93^c^	3.94^c^	0.070	<0.001	4.39^d^	8.71^b^	6.29^c^	8.43^b^	0.548	<0.001
DM	48.08^b^	30.21^e^	41.65^c^	38.48^d^	23.390	<0.001	47.02^b^	29.87^e^	39.06^c^	37.85^d^	22.060	<0.001	57.88^b^	35.63^d^	53.80^b^	46.95^c^	2.671	<0.001
DM loss (%)	NM						3.10^c^	1.65^c^	7.07^b^	2.11^c^	0.879	0.009	NM					
Nutrient composition, % DM basis																		
OM	93.61^b^	92.99^c^	93.92^b^	93.11^c^	0.114	0.002	93.38^b^	92.58^d^	92.98^c^	92.12^e^	0.144	<0.001	NM					
CP	14.20^b^	11.71^c^	11.60^c^	11.91^c^	0.059	0.003	13.41^b^	11.48^c^	13.63^b^	12.43^bc^	0.049	0.018	NM					
NDF	53.10^d^	58.73^b^	51.28^d^	55.59^c^	0.878	<0.001	49.34^c^	57.12^b^	56.06^b^	58.00^b^	1.121	0.003	NM					
ADF	27.04^d^	36.89^b^	30.51^c^	30.89^c^	1.116	0.001	24.89^d^	34.89^b^	33.30^c^	33.14^c^	1.189	<0.001	NM					
ADIN (% total N)	NM						4.43 ^bc^	5.19^b^	3.74^c^	4.78^b^	0.188	0.013	NM					
Starch	21.54^b^	9.14^d^	7.41^e^	10.76^c^	1.709	<0.001	19.20^b^	7.04^cd^	5.54^d^	8.30^c^	1.651	<0.001	NM					
WSC (mg/g DM)	4.66^e^	31.51^d^	74.60^b^	46.33^c^	7.305	<0.001	0.99^c^	2.52^c^	29.20^b^	12.01^bc^	7.821	0.031	NM					
Fermentation products, mg/g DM																		
Lactic acid			NM				44.64^e^	77.87^b^	64.56^c^	52.12^d^	3.913	<0.001	43.69^b^	6.60^d^	28.27^c^	5.86^d^	4.935	<0.001
Acetic acid			NM				28.92^b^	17.06^c^	16.85^c^	10.32^d^	1.895	<0.001	26.63^b^	1.19^c^	1.33^c^	0.87^c^	3.331	<0.001
Propionic acid			NM				0.09	ND	ND	ND	NA	NA	0.11	ND	ND	ND	0.015	NA
Butyric acid			NM				ND	0.01	ND	0.03	0.031	0.156	ND	0.03^b^	0.02^b^	0.02^b^	0.004	0.021
La:Ac ratio			NM				1.54^d^	4.56^b^	3.83^c^	5.05^b^	0.404	<0.001	1.64^c^	5.38^c^	21.42^b^	6.69^c^	2.487	0.002
Total VFAs			NM				29.09^b^	17.21^c^	17.08^c^	10.42^d^	1.892	<0.001	26.82^b^	1.35^cd^	1.93^c^	1.07^d^	3.314	<0.001
NH_3_-N	0.13^c^	0.10^d^	0.19^b^	0.11^cd^	0.011	0.001	2.34^b^	2.27^d^	2.13^c^	1.43^d^	0.109	<0.001	1.27^b^	0.22^d^	1.13^b^	0.61^c^	0.134	0.001
Microbiology, log_10_ CFU/g DM																		
Total bacteria	7.71^bc^	7.47^c^	7.97^b^	7.85^b^	0.085	0.0288	8.92^b^	8.40^c^	7.22^d^	8.05^c^	0.198	0.0011	7.75^d^	9.50^b^	8.78^c^	9.56^b^	0.223	<0.001
LAB	6.23	5.82	6.68	6.98	0.193	0.1267	8.98^b^	8.52^c^	7.83^d^	8.21^cd^	0.143	0.0017	7.78^c^	9.37^b^	9.28^b^	9.09^b^	0.216	0.003
Molds	6.22	6.06	5.99	6.12	0.051	0.4966	ND	ND	ND	ND	NA	NA	ND	8.00^b^	ND	7.18^c^	7.180	<0.001
Yeasts	7.1	7.04	7.09	7.03	0.021	0.4055	<1.00^c^	5.84^b^	6.34^b^	6.75^b^	0.481	0.0002	4.28^c^	8.82^b^	9.19^b^	8.63^b^	0.631	0.001
Aerobic Stability^a^	NM						NM						>336.00^d^	51.14^c^	142.91^b^	39.10^c^	17.379	0.001

^*^ Within a row, means with a different superscript (b,c,d,e) differ (*P* < 0.05)

^a^ Hours of aerobic exposure before a 2 °C rise in temperature was recorded in the silage mass after exposure to air

*Abbreviations*: *CP* crude protein, *NDF* neutral detergent fiber, *ADF* acid detergent fiber, *ADIN* acid detergent insoluble nitrogen, *WSC* water soluble carbohydrates, *NM* not measured, *ND* not determined, *NA* not available

The nutrient composition and fermentation profile of all silages differed (*P* < 0.05) after 90 days of fermentation (Table 1). At harvest, the OM of barley and triticale forages was higher than oat and intercropped forages, but after 90 days of ensiling OM levels differed (*P* < 0.001) among all small grain silages. Barley forage exhibited the highest level of CP, whereas after 90 days of ensiling oat silage exhibited the lowest (*P* < 0.05) CP of the silages and a higher ADIN (*P* < 0.05) content than triticale silage. Both barley and triticale forage exhibited lower (*P* < 0.05) levels of NDF than oats or intercropped forages, but after 90 days of ensiling only barley silage exhibited lower (*P* < 0.05) levels of NDF and ADF than other silages. Levels of WSC were noticeably lower (*P* < 0.001) in barley than other forages, but still differed among all forages. A higher (*P* < 0.05) WSC content was observed for triticale silage, while the WSC of barley silage remained low. The level of starch in barley was higher (*P* < 0.001) than other forages and silages, but declined in all silages by about 2 mg/g DM during ensiling. Lactic acid levels differed (*P* < 0.05) among all silage and were the highest for triticale silage and the lowest for barley silage. Acetic acid levels were highest (*P* < 0.05) in barley silage and the lowest in triticale silage. Propionic and butyric acids were either absent or only detected at trace levels.

Total bacterial populations were lower (*P* < 0.05) in oat forage as compared to triticale and intercropped forages (Table 1). Populations of LAB, molds and yeasts did not differ among fresh small grain forages. Compared to other silages, the total bacterial population (TB) was higher (*P* < 0.05) in barley and lower (*P* <0.05) in triticale, a pattern that was also observed for LAB populations. No molds were detected in any of the silages after 90 days of ensiling, with yeast populations ranging between 5.84 and 6.75 log_10_ CFU/g DM in oat, triticale and intercropped silages and were virtually absent in barley silage.

**Fig. 1:**
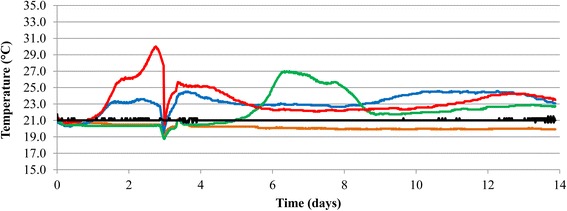
Silage temperature during aerobic exposure. Average temperature (°C) in barley (*orange*), oat (*blue*), triticale (*green*) and intercropped (*red*) silages (*n* = 3) and ambient temperature (*black*) (*n* = 2) recorded during 14 days of aerobic exposure

After aerobic exposure, the pH of oat, triticale and intercropped silages increased (*P* < 0.001) while the pH of barley silage remained largely unchanged (Table 1). The aerobic stability of barley was > 336 h, with no measurable increase in temperature over 14 days of aerobic exposure (Fig. 1). Oat and intercropped silage exhibited lower (*P* < 0.05) aerobic stability than triticale silage (Table 1), with temperature increases in these silages noticeable after 1 day of aerobic exposure (Fig. 1). With the exception of barley, levels of lactic acid declined (*P* < 0.05) in all silages, being the lowest in oat and intercropped silage. After 14 days of aerobic exposure, TB and LAB were lower (*P* < 0.05) in barley silage than in other small grain silages. Yeast populations in aerobically exposed barley silage remained at least 4 log_10_ CFU/g DM lower than (*P* < 0.001) in other silages. Molds were only detected in oat and intercropped silages after 14 days of aerobic exposure.

### Sequencing

A total of 8,937,418 and 3,959,537 reads were obtained for the bacterial and fungal microbiomes in silages, respectively. After bioinformatics analysis, a total of 526,210 bacterial sequences and 70,219 fungal sequences were classified.

#### Microbial silage diversity

Rarefaction curves for bacterial and fungal OTUs of silage samples at 97% identity are shown in Additional file 1: Figures S1a and S1b, respectively. Sequencing depth was insufficient to fully describe the diversity of the microbial populations in silage as rarefaction curves did not reach a clear plateau for either bacterial or fungal sequences. Bacterial diversity of the samples was observed to increase over the period of ensiling and aerobic exposure (i.e., 0, 90 and 104 days) as shown by differences in Chao1 and Shannon-Weiner indexes (*P* < 0.001, Additional file 2: Table S1). Barley silage was the exception as its bacterial diversity was similar to other silages after ensiling. This observation is supported by Additional file 3: Figure S2a which showed that the nature of the silage microbiome was more influenced by sampling time (Additional file 3: Figure S2a) than by silage type (Additional file 3: Figure S2c). Fungal Chao1 index did not differ (*P* > 0.05) among sampling times and exhibited similar richness among all silages. In contrast to bacteria, fungal communities appeared to be less influenced by sampling time or ensiling, although fungal sequences obtained prior to ensiling did tend to cluster together (Additional file 3: Figure S2b).

**Fig. 2:**
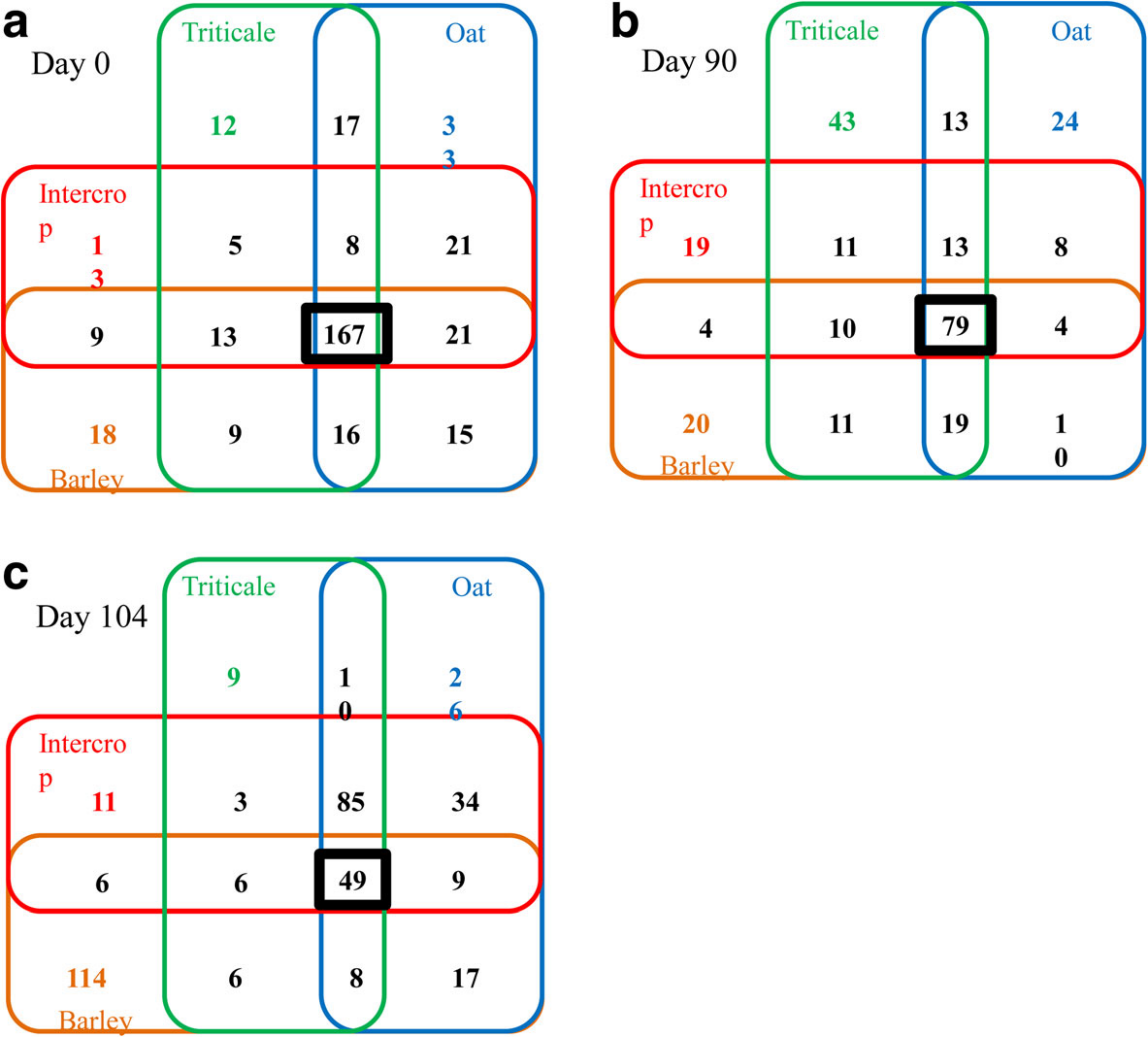
Four-way Venn diagram depicting unique bacterial OTUs in barley (*orange*), oat (*blue*), triticale (*green*) and intercropped (*red*) silages, or shared bacterial OTUs among silages at 0 days **a** 90 days **b** and 104 days **c**. Bacterial core microbiome OTUs number is displayed with *bold black frame*

A higher number of bacterial core microbiome OTUs were identified in fresh small grain forage (Fig. 2a) than in terminal (Fig. 2b) or aerobically exposed silage (Fig. 2c). The bacterial core microbiome of fresh forage was composed of 167 unique OTUs with the number of OTUs exclusively associated with each forage type ranging from 12 for triticale silage to 33 for oat silage (Fig. 2a). The diversity of the bacterial core microbiome declined after ensiling, being composed of 79 OTUs in terminal silage (Fig. 2b) and only 49 OTUs in aerobically exposed silage (Fig. 2c). After aerobic exposure, the number of bacterial OTUs in barley silage increased dramatically in spite of this silage being aerobically stable.

**Fig. 3:**
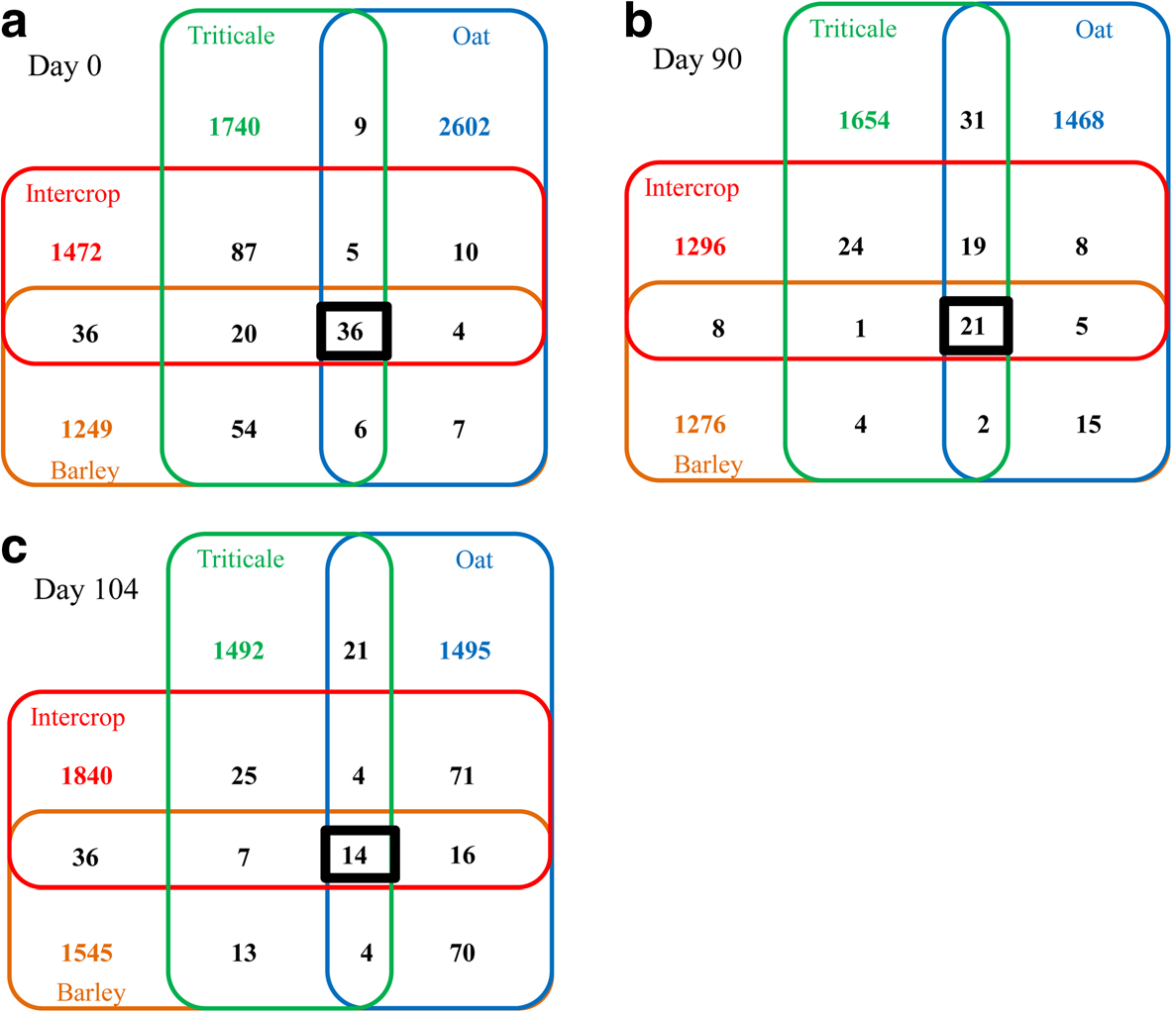
Four-way Venn diagram depicting unique fungal OTUs in barley (*orange*), oat (*blue*), triticale (*green*) and intercropped (*red*) silages, or shared bacterial OTUs among silages at 0 days **a** 90 days **b** and 104 days **c**. Fungal core microbiome OTUs number is displayed with *bold black frame*

The core fungal microbiome of silage consisted of 36 OTUs in fresh forage (Fig. 3a), and declined to 21 OTUs in terminal silage (Fig. 3b) and 14 OTUs in aerobically exposed silage (Fig. 3c). The highest number of shared OTUs occurred between intercropped and triticale forages (Fig. 3a) and between triticale and oat terminal silages (Fig. 3b) and between aerobically exposed intercropped and oat silages (Fig. 3c). With the exception of barley silage, the number of fungal OTUs for each crop decreased after ensiling, with aerobic exposure increasing the number of crop specific OTUs in intercropped, oat and barley silage.

#### Bacterial core microbiome during forage ensiling

**Fig. 4:**
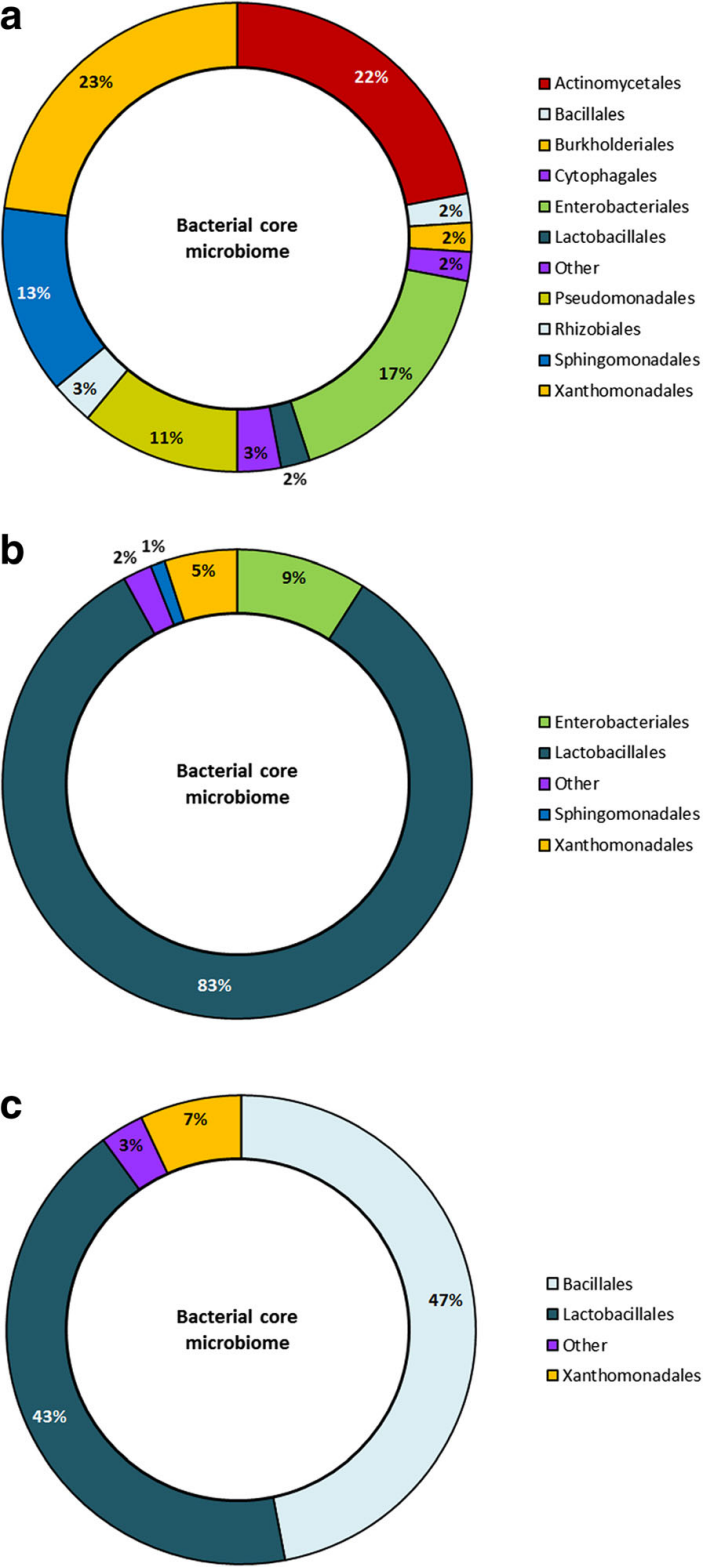
Taxonomic profile and relative abundance of the bacterial core microbiome after 0 days, fresh forage **a** 90 days, terminal silage **b** and terminal silage after 14 days of aerobic exposure **c**. OTUs were assigned at the order level

**Fig. 5:**
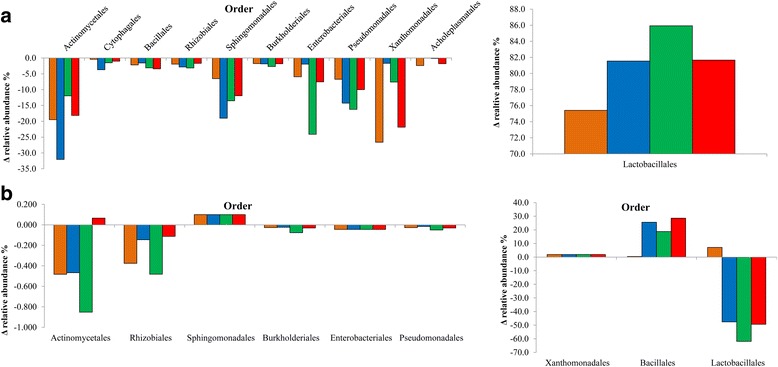
Bacterial core microbiome. **a** Average difference in the relative abundance of OTUs observed in bacterial core microbiome for barley (*orange*), oat (*blue*), triticale (*green*) and intercropped (*red*) fresh forages as compared to terminal silages (0 to 90 day) (*n* = 3). Bacterial orders that exhibited variation ≤ to 1% are not shown. Members of the Lactobacillales are presented separately to ease interpretation. **b** Average difference in the relative abundance of OTUs observed in bacterial core microbiome for barley (*orange*), oat (*blue*), triticale (*green*) and intercropped (*red*) silages after 14 days of aerobic exposure (90 to 104 days) (*n* = 3). Bacterial orders that exhibited variation ≤ to 0.05% in all silages are not shown. Xanthomonadales, Bacillales and Lactobacillales are presented separately to ease interpretation

Taxonomic profile of the bacterial core microbiome varied among fresh forage (Fig. 4a), terminal silage (Fig. 4b) and aerobically exposed silage (Fig. 4c). Up to 23 bacterial orders exhibited changes in relative abundance during ensiling, of which 12 orders accounted for less than 1.2% relative abundance variation in all silages (Fig. 5a). Among all silages, relative abundance of all bacterial orders, with the exception of the Lactobacillales, declined during the ensiling process (Fig. 4b). The decline in relative abundance of OTUs was the highest for the Actinomycetales as a decrease ranging from −11.2 to −32% was observed among silages, with the Sphingomonadales also showing a consistent decline in all silages (Fig. 5a). A strong reduction in the Xanthomonadales was also noted to occur during the ensiling of barley and intercropped silage, whereas Enterobacteriales strongly decreased in triticale silage (−24.1%). Most often the reduction in bacterial diversity during ensiling was also accompanied by a reduction in abundance. The most notable decreases in OTUs and relative abundance were observed in the Actinomycetales, Shingomonadales, Pseudomonadales and Xanthomonadales. Ten of the bacterial orders associated with core bacterial microbiome of fresh forage were no longer detected in terminal silage.

#### Bacterial core microbiome in aerobically exposed silage

The diversity of the bacterial microbiome continued to decline after aerobic exposure (Fig. 4c). During aerobic exposure, up to 13 orders from bacterial core microbiome exhibited differential abundance, of which 4 orders accounted for less than 0.05% of this variation among silages. The abundance of OTUs associated with Bacillales notably increased in oat, triticale and intercropped silages, but only a slight increase was observed in barley silage (Fig. 5b). The relative abundance of OTUs associated with Lactobacillales, also increased as a result of aerobic exposure of barley silage, whereas this order declined considerably in oat, triticale and intercropped silages (−49.3, −61.9 and −47.6%, respectively). The abundance of Xanthomonadales also increased in the core bacterial microbiome during aerobic exposure and this was linked to a higher diversity in all silages (Fig. 5b). With the exception of the Sphingomonadales and the Actinomycetales in intercropped silage (+0.07%), the abundance of all other bacterial orders declined after aerobic exposure. Bacillales represented almost half of the relative abundance of the core microbiome after aerobic exposure in all silages except barley (Fig. 4c).

Shared OTUs among aerobically exposed oat, triticale and intercropped silages were mainly members of the Bacillales order (98.82% of the number of OTUs observed) with only a single OTU belonging to the Streptococcaceae order being associated with these silages (Table 2). Among the Bacillales, the Bacillaceae represented 70.6% of the shared OTUs, whereas the Planococcaceae accounted for 22.4%. The OTUs found in aerobically exposed barley silage were mostly members of the Lactobacillales (27.2%).

**Table 2:** Taxonomic assignment, number and percent of shared and unique OTUs in oat, triticale and intercropped silages and barley silage respectively after 14 days of aerobic exposure

Item	Phylum	Class	Order	Family	OTU number	OTU %
Shared OTUs between oat, triticale and intercropped silages					85	100
	Firmicutes	Bacilli	Bacillales	Bacillaceae	60	70.59
	Firmicutes	Bacilli	Bacillales	NM	5	5.88
	Firmicutes	Bacilli	Bacillales	Planococcaceae	19	22.36
	Firmicutes	Bacilli	Lactobacillales	Streptococcaceae	1	1.18
Unique barley silage OTUs					114	100
	Actinobacteria	Actinobacteria	Actinomycetales	NS	14	12.28
	Actinobacteria	Thermoleophilia	Solirubrobacterales	NS	2	1.75
	Bacteroidetes	Cytophagia	Cytophagales	NS	2	1.75
	Bacteroidetes	Flavobacteriia	Flavobacteriales	NS	7	6.14
	Bacteroidetes	Sphingobacteriia	Sphingobacteriales	NS	7	6.14
	Firmicutes	Bacilli	Bacillales	NS	2	1.75
	Firmicutes	Bacilli	Lactobacillales	NS	31	27.19
	Proteobacteria	α-proteobacteria	Rhizobiales	NS	11	9.65
	Proteobacteria	α-proteobacteria	Rhodobacterales	NS	3	2.63
	Proteobacteria	α-proteobacteria	Sphingomonadales	NS	6	5.26
	Proteobacteria	β-proteobacteria	Burkholderiales	NS	9	7.89
	Proteobacteria	γ-proteobacteria	Alteromonadales	NS	1	0.88
	Proteobacteria	γ-proteobacteria	Enterobacteriales	NS	6	5.26
	Proteobacteria	γ-proteobacteria	Pseudomonadales	NS	9	7.89
	Proteobacteria	γ-proteobacteria	Xanthomonadales	NS	3	2.63
	Tenericutes	Mollicutes	Acholeplasmatales	NS	1	0.88

*Abbreviations*: *NM* not measured, *NS* non shown

#### Fungal core microbiome during forage ensiling

**Fig. 6:**
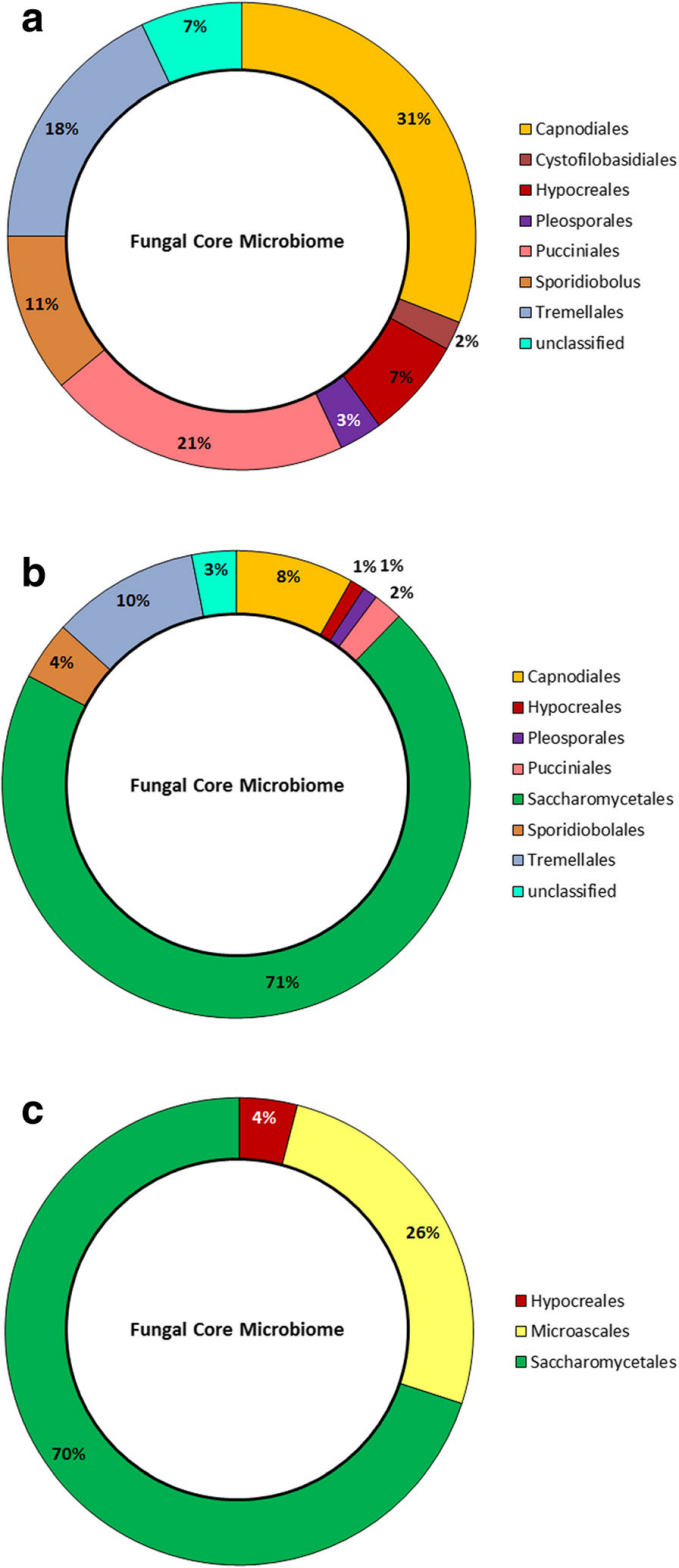
Taxonomic profile and relative abundance of the fungal core microbiome after 0 days, fresh forage **a** 90 days, terminal silage **b** and terminal silage after 14 days of aerobic exposure **c**. OTUs were assigned at the order level

**Fig. 7:**
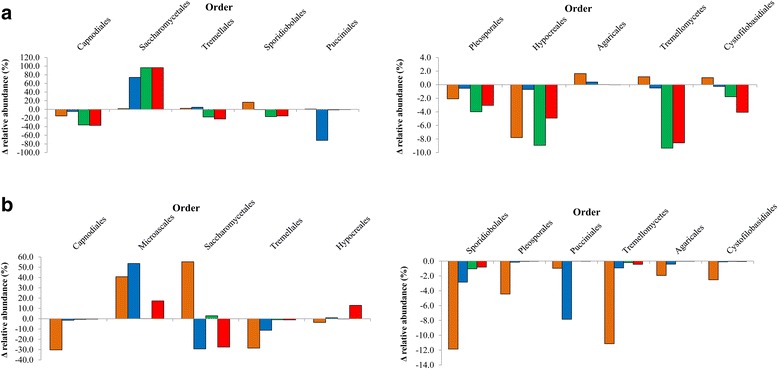
Fungal core microbiome during ensiling. **a** Average difference in the relative abundance of OTUs observed in fungal core microbiome for barley (*orange*), oat (*blue*), triticale (*green*) and intercropped (*red*) forages as compared to terminal silages (0 to 90 day) (*n* = 3). Fungal orders that exhibited variation ≤ 1% are not shown. **b** Average difference in the number of OTUs observed in fungal core microbiome for barley (*orange*), oat (*blue*), triticale (*green*) and intercropped (*red*) silages after aerobic exposure for 14 days (from 90 to 104 days) (*n* = 3). Fungal orders that exhibited variation ≤ 0.7% are not shown

The diversity of fungi also tended to decline during the ensiling process (Fig. 6a and b). In total, 19 fungal orders were altered by the ensiling process, of which 9 orders accounted for less than 1% variation in relative abundance in all silages (Fig. 7a). The changes in the fungal population observed during the ensiling of barley differed from that of other silages as the relative abundance of OTUs associated with members of the Sporidiobolales, Pucciniales, Tremellomycetes and Cystofilobasidiales increased (Fig. 7a). During ensiling, numbers of OTUs in the Tremellales and Agaricales increased in barley and oat silages. The number of OTUs associated with Capnodiales, identified as members of the *Cladosporium* (Additional files 4 and 5: Figures S3 and S4) also declined, representing only 8% of the fungal core microbiome after ensiling (Fig. 6b). Saccharomycetales represented almost 70% of the fungal core microbiome in terminal silage, with most of the OTUs being associated with *Kazachtania* (61%) and *Pichia* (8%). (Additional files 4 and 5: Figures S3 and S4). The fungal core microbiome was not consistent among terminal silages as the relative abundance of Saccharomycetales increased by 1.52% in barley silage as compared to an increase of 73.95 to 96.37% OTUs in other silages (Fig. 7a). In contrast, Pleosporales slightly decreased in relative abundance in all silages. Orders such as Eurotiales and Chaetothyriales represented a small proportion of the fungal core microbiome with few differences between fresh small grain forages and terminal silages (data not shown).

#### Fungal core microbiome in aerobically exposed silage

In general, the diversity of the fungal core microbiome decreased during aerobic exposure (Fig. 6c). As many as 7 fungal orders that were detected in terminal silage were no longer detected in aerobically exposed silage. Fifteen orders of the fungal core microbiome underwent changes in relative abundance after aerobic exposure of which 4 orders accounted for less than 0.7% variation in all silages (Fig. 7b). The greatest decline was observed in barley and oat silages, but the Sporidiobolales, Pleosporales, Puccinales, Tremellomycetes, Agaricales and Cystofilobasidiales also declined in all silages after aerobic exposure (Fig. 7b). An increase in diversity and OTUs associated with the Microascales, occurred because of an increase in the *Corollospora* (Additional file 6: Figure S5), mainly in barley, oat and intercropped silages (Fig. 7b). The relative abundance of OTUs belonging to Saccharomycetales also increased by 55.33 and 2.85% in barley and triticale silages respectively, whereas these populations decreased in oat and intercropped silages (−29.35 and −27.51%). Saccharomycetales still represented 70% of the fungal core genome after aerobic exposure, primarily due to the high abundance of *Kazachstania* (62%) and *Pichia* (8%) (Additional file 6: Figure S5). An increase of 12.92% in relative abundance of Hypocreales was also observed in intercropped silage (Fig. 7b).

## Discussion

Present knowledge of the silage microbiome has been mainly garnered through culture dependant methods which are known to underestimate microbial diversity. Microbial epiphytic population of plants may affect the quality and productivity of agricultural crops [46]. In this study, rDNA sequencing was used to elucidate the dynamics of the microbial ecology of small grain silages by studying the variability over time of the silage microbial core genome. While silages can have their own distinct microbial profile, the true ‘core microbiome’ can be defined as those microbes that are common to all small grain cereals.

### Silage characteristics

As observed by McCartney and Vaage [6], differences in stage of maturity among forages at harvest influences the composition and quality of ensiled crops. In this study, all small grain cereal silages, with the exception of barley silage, obtained a pH of < 4.0 after 90 days of ensiling. Barley silage was harvested at the firm-dough stage and exhibited a high DM and low WSC content, suggesting it was overly mature at harvest [47]. Compared to the other silages, levels of lactic acid were notably lower and acetic acid higher in barley silage. This indicates a strong homofermentative acid fermentation in oat, triticale and intercropped silages, whereas a switch from homo- to heterofermentation likely occurred during the ensiling of barley. Only traces of butyric acid were found in oat and intercropped silages, indicating that all silages were well conserved and that growth of spoilage microorganisms was limited. These observations were in accordance with the microbial counts observed, as no molds were detected at opening, yeasts were only cultured from oat, triticale and intercropped silages and numbers of LAB exceeded 7.83 log_10_ CFU/g DM in all small grain silages.

### Core microbiome during ensiling of small grain cereal forages

The number of different OTUs identified were higher for fungal than for the bacterial core microbiome, a result that can be explained by differences in the databases and analyzes used for taxonomic assignments. Bacterial taxonomic assignments relied on the Greengenes database and a RDP classifier with a depth taxonomic OTU threshold and similarity cut off for taxonomic clustering. As the RDP classifier is not designed to process 18S rDNA sequences, the Silva database was used for fungal identification and only a similarity cut off was applied for taxonomic assignment. It might be possible that OTUs belonging to the same lineage (i.e., one hit identified at the family level and the other at the genus level for example) were identified as different OTUs through 18S rDNA analysis, increasing the final number of estimated fungal OTUs, a bias that likely did not occur during bacterial analysis. The validity of these taxonomic assignments was still acceptable as bacterial and fungal communities were never compared directly. Bacterial communities in silage varied over time as shown by PCoA. The greatest microbial diversity in the core bacterial microbiome of barley, oat, triticale and intercropped silages was observed to be associated with the fresh forage. The bacterial population observed at that time was dominated by Proteobacteria, mainly members of the Xanthomonadales, Pseudomonadales Enterobacteriales and Sphingomonadales. Actinobacteria also accounted for a significant portion of the core bacterial microbiome at ensiling. These results are in accordance with literature as α- and γ-Proteobacteria were found to be dominant members of the bacterial microbiome on maize leaves [48]. Leaves of field-grown barley (cultivar Sumo) were found to be largely colonized by Pseudomonadales, although Enterobacteriales such as *Erwinia* were also detected [49]. *Azospirillum* and *Agrobacterium* belonging to α-proteobacteria have also been shown to be associated with oat leaves [50]. It is noteworthy that the initial proportion of Lactobacillales in the core bacterial microbiome of small grain forages was low. This is a reflection of the limited LAB population observed in the field (only 26 different OTUs belonging to Lactobacillales were identified in fresh forages), but also the diversity of epiphytic LAB found among the different crops, precluding some of the OTUs as contributors to the core microbiome of fresh small grain forages. Others have found that LAB constitute a small proportion of the epiphytic bacterial population in most forages as Mogodiniyai Kasmaei et al. [51] observed a relative abundance of initial LAB population in green grass, red clover and maize ranging from 0.18 to 3.77%.

Little information is available with regard to the epiphytic fungal communities in silage, although the presence and growth of mycotoxigenic fungi has been extensively studied [52]. Barley and oat forages can be heavily colonized by mycotoxigenic fungi such as *Fusarium* which belongs to the Hypocreales [53]. *Fusarium*, *Aspergillus*, *Penicillium, Mucor, Rhizopus, Cladosporium,* and *Absidia* were identified in a 4-year survey of small grain cereals including barley, oats and triticale in eastern Romania [54]. We also identified the presence of potential mycotoxigenic fungi within the core microbiome of small grain silages such as members of the *Cladosporium* of the Capnodiales. Although we did not specifically identify *Fusarium,* fungi belonging to the Hypocreales were found in high quantities in barley, intercropped and triticale silages.

Yeasts are considered one of the main fungal colonizers of wheat leaves [55] and several genera have been identified in barley including *Sporobolomyces* and *Cryptococcus* [56]. The fungal core microbiome at ensiling was dominated by members of the Basidiomycota phylum, mainly members of the Pucciniales and Tremellales, such as *Cryptococcus* or Sporodiobolales, such as *Sporidiobolus*. Pucciniales OTUs were identified primarily as *Puccinia,* which was more prominent in oat silage. *Puccinia* are known to be responsible for rust, a disease that results in significant losses in small grain cereal crops [57]. Stripe rust caused by *Puccinia striiformis* has been frequently found in wheat, barley and triticale in Alberta [58].

The main microbial changes that occur during ensiling are well known from a culturable perspective [59] and have been recently reviewed [13]. Aerobic microflora remain metabolically active in silage for a few days until oxygen is depleted and acidification inhibits microbial metabolism. In properly ensiled forage, facultative anaerobic bacteria gradually decrease silage pH, promoting the growth of acid-tolerant LAB, which typically dominate the bacterial microbiome of terminal silage. Numbers of viable LAB tend to decrease during storage with the exception of some heterofermentative species such as *Lactobacillus buchneri,* which convert lactic acid into acetic acid and have been identified in corn silage a month after fermentation [60]. Culture independent techniques such as DGGE, T-RFLP or LH-PCR have been used to characterized silage bacterial communities and shown a predominance of *Lactobacillus*, *Lactococcus* and *Pediococcus* during ensiling [61, 62]. These observations are in accordance with our results as the core bacterial microbiome after 90 day of fermentation in silage showed reduced diversity and was largely dominated by members of the Lactobacillales. Using 16S pyrosequencing and metagenomic analysis, Eikmeyer et al. [23] observed a predominance of bacteria from the phylum Firmicutes in grass silage after 58 days of ensiling. Members of the Lactobacillales, including *Lactococcus*, *Lactobacillus* and *Weissella* accounted for 80% of relative bacterial abundance, a result similar to our observations. Bacterial population sequencing in ryegrass, whole crop corn and alfalfa silages also illustrated the intensive selection process that occurs during ensiling as bacterial diversity in initial forages dramatically declined with Leuconostocaceae and Lactobacillaceae representing about 70 to 95% of the bacterial population in terminal silages [63]. Bao et al. [64] used single molecule real-time sequencing technology to study the bacterial population in inoculated alfalfa silages. They observed an expansion in the LAB population during ensiling with inoculated LAB species dominating, but the extent of this domination could not be ascertained as uninoculated control silage was not included in the study. The shift in bacterial microbiome from Proteobacteria and Actinobacteria to Firmicutes is key to ensuring the conservation of small grain silages. Members of the Proteobacteria, Xanthomonadales, Sphingomonadales, Enterobacteriales and Actinomycetales comprised the remainder of the microbiome in small grain silages. McEniry et al. [65], observed that the population of Enterobacteria associated with grass silage declined within 48 h after ensiling.

Butyric acid producing bacteria (BAB) such as Clostridia and *Bacillus* are considered undesirable in silage. These bacteria promote the growth of less acid-tolerant spoilage microorganisms and can contaminate milk and milk products with BAB spores, resulting in significant economic losses [12]. Clostridia was not detected within the core microbiome of terminal silage in this study and only trace amounts of butyric acid were observed in oat and intercropped silages. Our study used mini-silos, which can create an ensiling environment that is likely more homogeneous than that achieved in field scale bunker or upright silos. These differences in ensiling environment may also influence the nature of the microbiome that establishes in terminal silage. Kraut-Cohen et al. [66] defined bacterial populations in bunker silos using Ion Torrent PGM sequencing technology, as being “preserved” regions in the center and “spoiled” regions along the edges, with the composition of the bacterial microbiome clearly differing between these sampling sites. Although numbers of total bacteria and LAB were the lowest in triticale silages after ensiling, a higher number of specific OTUs were observed in this silage. This suggests that some of the microorganisms associated with triticale silage were nonculturable using plating techniques within the laboratory, an observation that may also account for the slightly higher numbers of LAB as compared to total bacteria in this silage.

The limitations of using culture-based approaches to document the impact of fungal populations in small grain silages is illustrated by our inability to culture molds and to only detect yeast in oat, triticale and intercropped silages. Use of sequencing to characterize the fungal microbiome identified 21 OTUs within the core microbiome. The number of fungal OTUs did decline with ensiling, reflecting the acidification and transition from an aerobic to an anaerobic environment. Although most fungi are strict aerobes, a few filamentous fungi and several yeasts are capable of fermentative growth and can survive the ensiling process. The strongest decrease in members of the fungal core microbiome was observed for *Cladosporium* a member of the Capnodiales. Saccharomycetales dominated the core fungal microbiome in all silages, with the exception of barley silage. The initial low level of WSC in barley silage (4.66 mg/g DM) may have limited the growth of Saccharomycetales as compared to the other silages. Saccharomycetales including *Candida* and *Issatchenkia* have been shown to be associated with barley silage using culture techniques, but no filamentous fungi were observed [67]. Members of the Sporidiobolales and Tremellales were also found to be minor components of the fungal core microbiome in small grain silages. As in our study, others have used molecular techniques to confirm that members of the Saccharomycetales including *Candida*, *Pichia* or *Saccharomyces* are the predominant fungi associated with terminal silage [68–70].

### Core microbiome in aerobically exposed silage

The diversity of the core bacterial microbiome decreased during aerobic exposure. Oat, triticale and intercropped silages exhibited similar bacterial diversity, whereas a high number of specific OTUs were observed in barley silage. These observations are in accordance with the significant lower counts of total bacterial and LAB populations in barley silage as compared to other silages. However, the number of OTUs associated with Lactobacillales increased in aerobically exposed barley. It can be hypothesized that some of the Lactobacillales species were in a viable, but nonculturable state, with these populations increasing during aerobic exposure. This is in accordance with the high DM content observed at opening in barley silage. This low level of moisture likely prevented a rapid growth of many microorganisms and limited further silage spoilage during aerobic exposure as indicated by its low temperature profile and a lack of pH increase. In contrast, the temperature of oat, triticale and intercropped silages increased and high pH values were reached in these silages after aerobic exposure. Exposure to oxygen is known to promote yeast growth and lactic acid consumption. Contrary to the observations in other silages, diversity of Lactobacillales and Xanthomonadales increased in barley silage during aerobic exposure and far fewer OTUs were assigned to Bacillales as compared to other silages. The Lactobacillales present in barley silage during ensiling may have proliferated at the beginning of the aerobic exposure despite the high DM content observed. Such a scenario in aerobically exposed silage could lead to a slower increase in silage pH and thus limit the growth of spoilage microorganisms.

*Listeria* is considered as one of the main pathogenic bacteria in silage [71] as it is responsible for listeriosis which can result in encephalitis, abortion, septicemia and even death in animals and humans [72]. Although *Listeria* belongs to the Bacillales family, which increased in most silages during aerobic exposure, none of the observed OTUs were identified as *Listeria* at the sequencing depth used in this study. Production of butyric acid in silage arises primarily from *Clostridium* and *Bacillus* and can result in reductions in silage intake [12, 73]. Although these undesirable bacteria were not formally identified at the species level, many OTUs were assigned to *Bacillus* after 14 days of aerobic exposure in all silages. These observations confirm the significant role of Bacillales in the deterioration of small grain silage during aerobic exposure.

Yeasts belonging to the Saccharomycetales such as *Issatchenkia* sp., *Candida* sp. and *Saccharomyces* sp. have been shown to be associated with barley silage during aerobic exposure [67]. In this study, the Saccharomycetales order dominated the core fungal microbiome of silage before and after aerobic exposure. Most of these OTUs were assigned to *Kazachstania* and *Pichia*. Depending on the teleomorph or anamorph state of the cell, some *Kazachstania* can also be identified as *Candida* species [74]. It can thus be hypothesized that the high proportion of Saccharomycetales observed in the core microbiome after aerobic exposure is likely reflective of the main spoilage genera, *Candida* and *Pichia* [75]. Intercropped and oat silages contained the lowest concentration of acetic acid, which is known to improve aerobic stability of silage by inhibiting the growth of fungi [9, 76]. These two silages exhibited similar temperature profiles during aerobic exposure and the poorest aerobic stability. Aerobic exposure in these silages was linked with a decrease in the diversity and abundance of the Saccharomycetales and an increase in the diversity of Microascales and Hypocreales. This is in accordance with the literature as the succession of fungal species during aerobic exposure of silage is typically initiated by yeasts with the increase in pH allowing less acid-tolerant spoilage microorganisms to proliferate [77]. According to the temperature profile, spoilage was delayed in triticale silage as the switch from Saccharomycetales to other spoilage fungi did not appear to occur even after 14 days of aerobic exposure. A clear link cannot be made between Saccharomycetales abundance and temperature in barley silage as there was no evidence of deterioration during aerobic exposure. As a result, Saccharomycetales diversity and relative abundance increased as well as for the Microascales. Microascales have been found in composted silage using Illumina Miseq sequencing [23] and the filamentous fungal species *Microascus brevicaulis* has been identified in barley silage after exposure to air [67]. Fungi such as *Aspergillus flavus* and *Penicillium verrucosum*, belonging to the Eurotiales are known to be among the main post-harvest mycotoxin producers and are thus considered undesirable in silage [78]. Their presence has been previously observed in barley silage several decades ago [79]. OTUs assigned to Eurotiales were found in the fungal core microbiome although their relative abundance was only of 0.2%, and they were associated solely with oat silage.

## Conclusion

To our knowledge, this study is the first to use next-generation sequencing to define the core bacterial and fungal microbiome of small grain silages. Using rDNA gene sequencing through Illumina technology highlighted the role of the main bacterial and fungal orders as well as the microbial succession that occured during the ensiling process. As expected, a high dominance of Lactobacillales species was observed during storage and growth of spoilage microorganisms was noted during aerobic exposure. This work is also the first to study the core fungal microbiome depicting fungal communities succession during the complex process of ensiling (from fresh forage to aerobically exposed silage). This paper has highligted the dominance of yeasts belonging to the Saccharomycetale order in the core microbiome of small grain silages after ensiling. Aerobic exposure was characterized by an increase of OTUs belonging to the Hypocreales, frequently associated with saprophytic fungi, indicating a decaying process in silage, or with plant pathogenic fungi such as *Sarocladium* sp.. Using next generation sequencing to define the microbial ecology of silage could facilitate the development of additives that could act synsergistically with defined populations to improve the quality and aerobic stability of small grain cereal silages.

## Additional files

Additional file 1: Figure S1. Rarefaction curves. Rarefaction curves depicting the effect of 3% dissimilarity on the number of bacterial (A) or fungal (B) OTUs observed for barley (orange), oat (blue), triticale (green) and intercropped (red) silages. (TIF 1473 kb)
Additional file 2: Table S1. Bacterial and Fungal Diversity Index. **Table S2.** Mapping File used for MOTHUR pipeline. (DOCX 18 kb)
Additional file 3: Figure S2. PCo analysis. Principal coordinates analysis for bacterial (left) and fungal (right) communities according to sampling time; fresh forage, terminal silage, aerobically exposed silage (A and B) and silage type (C and D). (TIF 822 kb)
Additional file 4: Figure S3. Taxonomic profile and relative abundance of the fungal core microbiome of fresh forage. OTUs were assigned at the genus level. (TIF 559 kb)
Additional file 5: Figure S4. Taxonomic profile and relative abundance of the fungal core microbiome after ensiling (90 day). OTUs were assigned at the genus level. (TIF 458 kb)
Additional file 6: Figure S5. Taxonomic profile and relative abundance of the fungal core microbiome after aerobic exposure (14 days). OTUs were assigned at the genus level. (TIF 427 kb)

## Abbreviations

ADF: Acid detergent fibre
ADIN: Acid detergent insoluble nitrogen
BAB: Butyric acid bacteria
CFU: Colony forming units
CP: Crude protein
DDGE: Denaturing gradient gel electropheriss
DM: Dry matter
DNA: Deoxyribonucleic acid
EDTA: Ethylenediaminetetraacetate
K: Potassium
LAB: Lactic acid bacteria
LH-PCR: Length heterogeneity polymerase chain reaction
MRS: Man Rogosa Sharpe
N: Nitrogen
NA: Nutrient agar
NDF: Neutral detergent fibre
OM: Organic matter
OTU: Operational taxonomic unit
P: Phosphorus
PCoA: Principal component analysis
rDNA: Ribosomal DNA
RDP: Ribosomal database project
SDA: Sabouraud dextrose agar
SSU: Small sub unit
TB: Total bacteria
T-RFLP: Terminal restriction fragment length polymorphism
WSC: Water soluble carbohydrate
