# Supplementary figures and images for: Bacterial and fungal core microbiomes associated with small grain silages during ensiling and aerobic spoilage
Source: BMC Microbiol. 2017 Mar 3;17:50. doi: 10.1186/s12866-017-0947-0 (PMC5335695; doi:10.1186/s12866-017-0947-0)

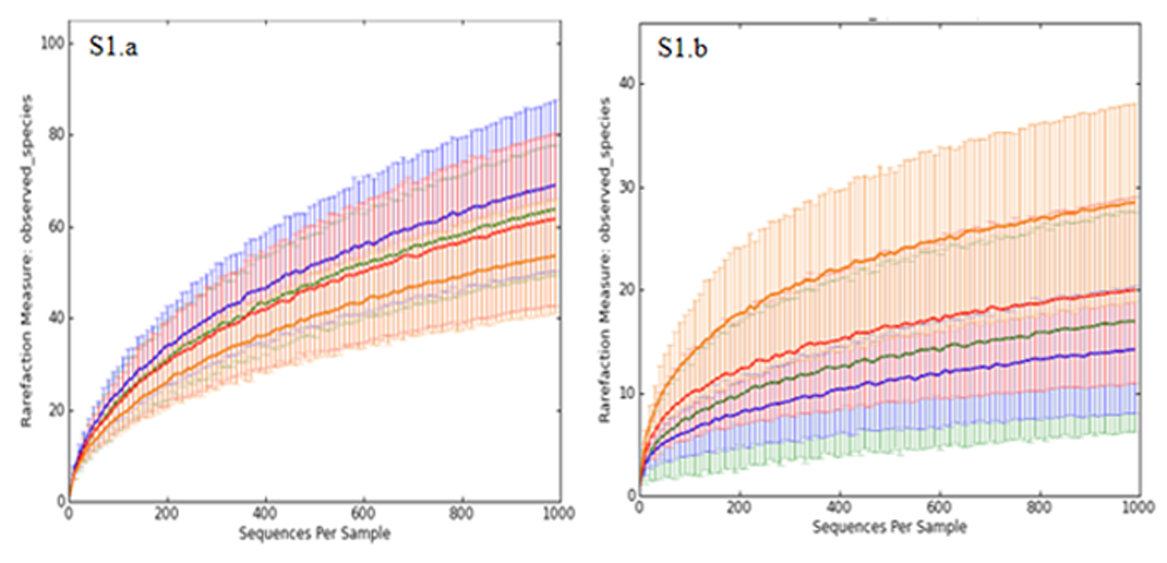

Supplement: Additional file 1: Figure S1. — Rarefaction curves. Rarefaction curves depicting the effect of 3% dissimilarity on the number of bacterial (A) or fungal (B) OTUs observed for barley (orange), oat (blue), triticale (green) and intercropped (red) silages. (TIF 1473 kb) [file 12866_2017_947_MOESM1_ESM.tif]

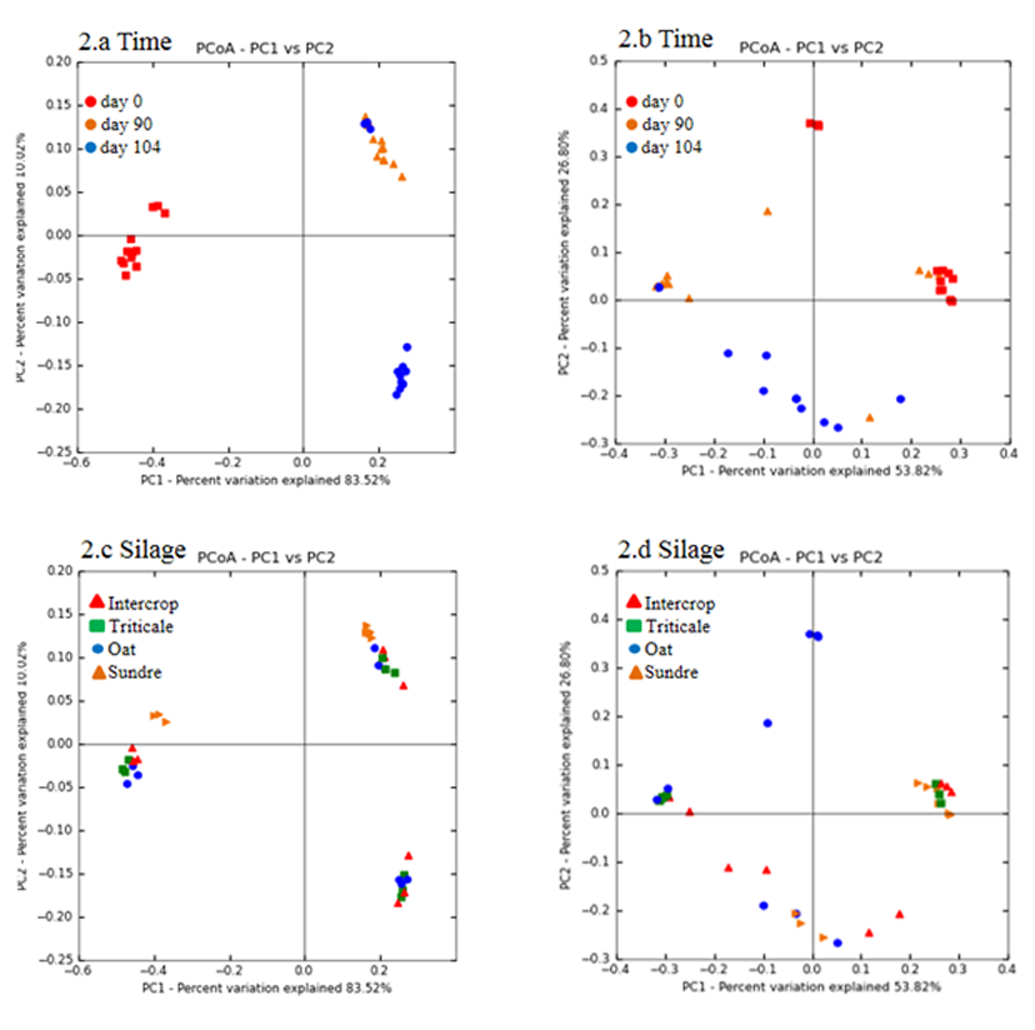

Supplement: Additional file 3: Figure S2. — PCo analysis. Principal coordinates analysis for bacterial (left) and fungal (right) communities according to sampling time; fresh forage, terminal silage, aerobically exposed silage (A and B) and silage type (C and D). (TIF 822 kb) [file 12866_2017_947_MOESM3_ESM.tif]

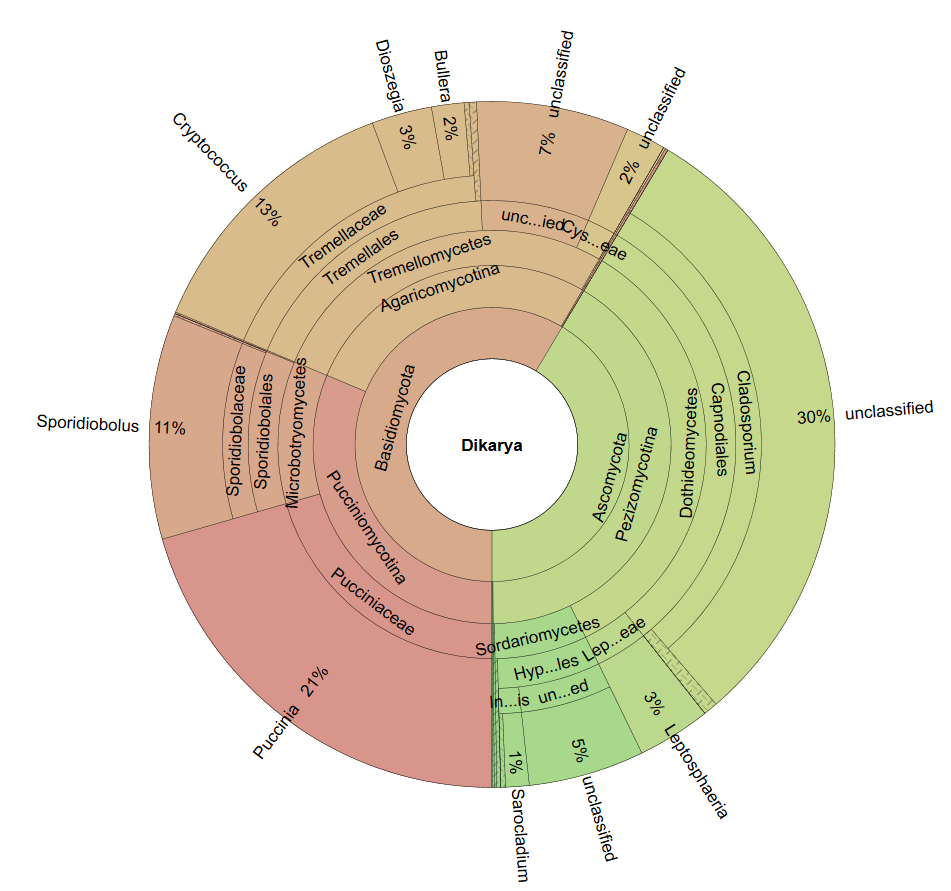

Supplement: Additional file 4: Figure S3. — Taxonomic profile and relative abundance of the fungal core microbiome of fresh forage. OTUs were assigned at the genus level. (TIF 559 kb) [file 12866_2017_947_MOESM4_ESM.tif]

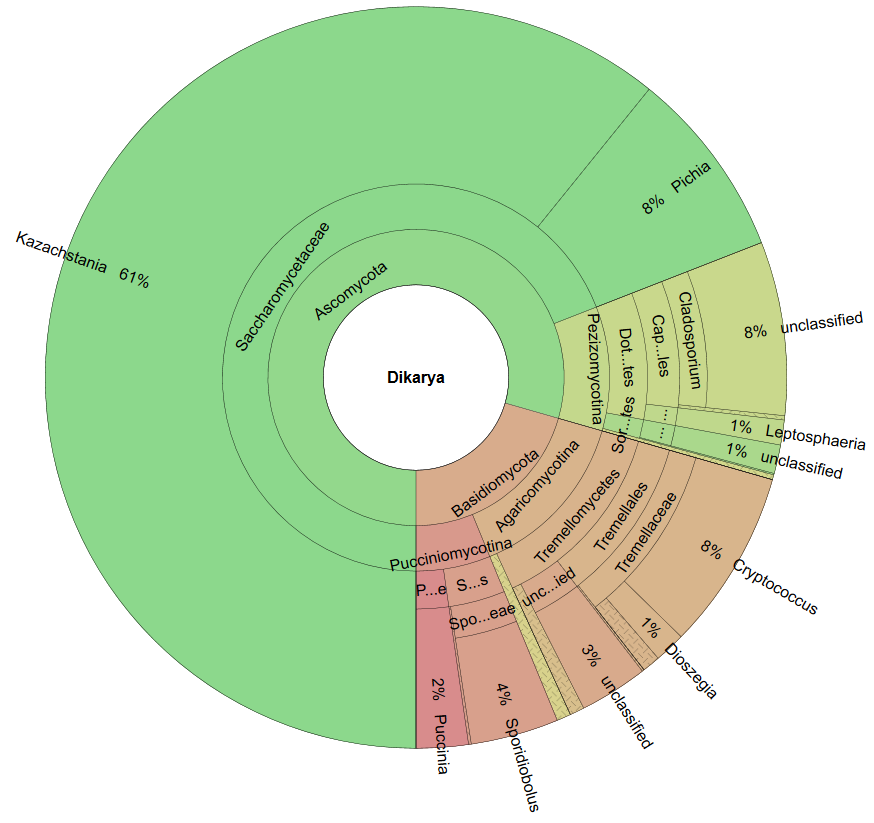

Supplement: Additional file 5: Figure S4. — Taxonomic profile and relative abundance of the fungal core microbiome after ensiling (90 day). OTUs were assigned at the genus level. (TIF 458 kb) [file 12866_2017_947_MOESM5_ESM.tif]

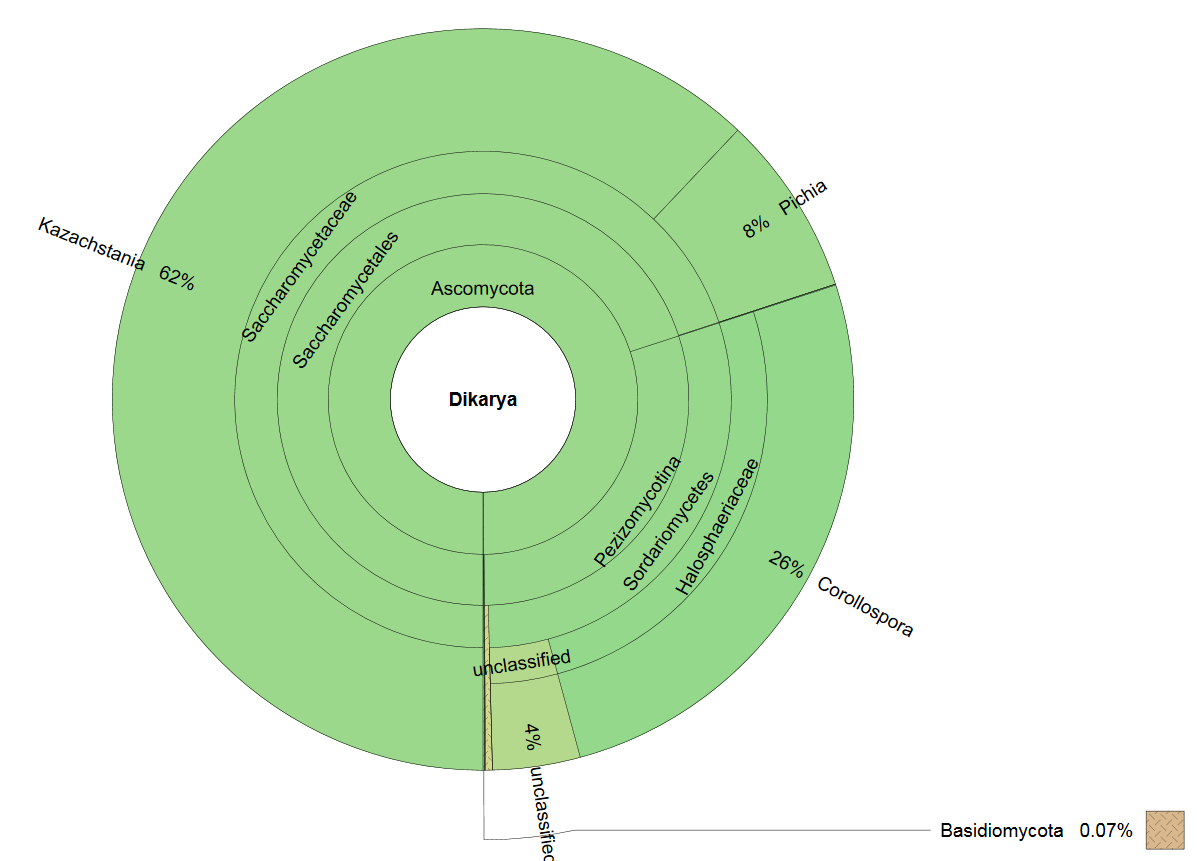

Supplement: Additional file 6: Figure S5. — Taxonomic profile and relative abundance of the fungal core microbiome after aerobic exposure (14 days). OTUs were assigned at the genus level. (TIF 427 kb) [file 12866_2017_947_MOESM6_ESM.tif]
