# Supplementary material for: Bacterial and fungal core microbiomes associated with small grain silages during ensiling and aerobic spoilage
Source: BMC Microbiol. 2017 Mar 3;17:50. doi: 10.1186/s12866-017-0947-0 (PMC5335695; doi:10.1186/s12866-017-0947-0)
Supplement: Additional file 2: Table S1. — Bacterial and Fungal Diversity Index. Table S2. Mapping File used for MOTHUR pipeline. (DOCX 18 kb) [file 12866_2017_947_MOESM2_ESM.docx]

**Additional Files**

Table S1. Bacterial and Fungal Diversity Index

| **Kingdom** | **Index** | **Barley** | | | **Intercropped** | | | **Oat** | | | **Triticale** | | | **SEM** | **P** |
| --- | --- | --- | --- | --- | --- | --- | --- | --- | --- | --- | --- | --- | --- | --- | --- |
|  |  | **0** | **90** | **114** | **0** | **90** | **114** | **0** | **90** | **114** | **0** | **90** | **114** |  |  |
| Bacteria | Chao1 | 2967^a^ | 5698^be^ | 5763^ce^ | 2219^a^ | 5571^bc^ | 10 759^d^ | 2595^a^ | 5422^bc^ | 9736^d^ | 2020^a^ | 5512^bc^ | 9798^d^ | 877.18 | < 0.001 |
|  | Shannon-Weiner | 4.93^a^ | 6.19^b^ | 6.18^b^ | 4.09^c^ | 6.17^b^ | 8.79^d^ | 3.93^c^ | 5.86^b^ | 8.33^d^ | 3.46^c^ | 6.03^b^ | 6.01^d^ | 0.51 | < 0.001 |
| Fungi | Chao1 | 6105 | 3682 | 5704 | 5963 | 5276 | 7657 | 6965 | 7286 | 5181 | 6072 | 5465 | 5414 | 307.83 | 0.088 |
|  | Shannon-Weiner | 4.21^ab^ | 4.64^ac^ | 3.19^ab^ | 4.43^acd^ | 2.60^bd^ | 3.42^ab^ | 2.72^ab^ | 2.72^ab^ | 2.99^ab^ | 4.32^cd^ | 2.12^be^ | 2.14^e^ | 0.261 | < 0.001 |

Diversity Index of Bacterial and Fungal populations of barley, oat, triticale and intercropped silages during ensiling process (n=3).

^1^ Within a row, means without a common superscript differ (*P* < 0.05).

Table S2: Mapping File used for MOTHUR pipeline

| Item | Denomination | Explanation |
| --- | --- | --- |
| Sequencing | A | Bacterial sequencing |
|  | B | Fungal sequencing |
| Forage type | S | Barley |
|  | I | Intercropped |
|  | O | Oat |
|  | T | Triticale |
| Sampling day | 0 | Ensiling |
|  | 90 | Terminal silage |
|  | 104 | Aerobic exposure |

As an example, paired-ends reads obtained with MiSeq Illumina sequencing were identified as follow: AS01.1 and AS01.2 for bacterial sequencing of barley silage at 0 d, forward and reverse reads 1 and 2 respectively.

Additional File 2: Figure S1. Rarefaction curves. Rarefaction curves depicting the effect of 3 % dissimilarity on the number of bacterial (**A**) or fungal (**B**) OTUs observed for barley (orange), oat (blue), triticale (green) and intercropped (red) silages.

Additional File 3: Figure S2. PCo analysis. Principal coordinates analysis for bacterial (left) and fungal (right) communities according to sampling time; fresh forage, terminal silage, aerobically exposed silage (**A** and **B**) and silage type (**C** and **D**).

Additional File 4: Figure S3. Taxonomic profile and relative abundance of the fungal core microbiome of fresh forage. OTUs were assigned at the genus level.

Additional File 5: Figure S4. Taxonomic profile and relative abundance of the fungal core microbiome after ensiling (90 d). OTUs were assigned at the genus level.

Additional File 6: Figure S5. Taxonomic profile and relative abundance of the fungal core microbiome after aerobic exposure (14 d). OTUs were assigned at the genus level.
